# Frames as visual links between paintings and the museum environment: an analysis of statistical image properties

**DOI:** 10.3389/fpsyg.2013.00831

**Published:** 2013-11-08

**Authors:** Christoph Redies, Franziska Groß

**Affiliations:** Experimental Aesthetics Group, Institute of Anatomy I, University of Jena School of Medicine, Jena University HospitalJena, Germany

**Keywords:** art perception, experimental aesthetics, self-similarity, complexity, statistical image properties, museum paintings

## Abstract

Frames provide a visual link between artworks and their surround. We asked how image properties change as an observer zooms out from viewing a painting alone, to viewing the painting with its frame and, finally, the framed painting in its museum environment (museum scene). To address this question, we determined three higher-order image properties that are based on histograms of oriented luminance gradients. First, complexity was measured as the sum of the strengths of all gradients in the image. Second, we determined the self-similarity of histograms of the orientated gradients at different levels of spatial analysis. Third, we analyzed how much gradient strength varied across orientations (anisotropy). Results were obtained for three art museums that exhibited paintings from three major periods of Western art. In all three museums, the mean complexity of the frames was higher than that of the paintings or the museum scenes. Frames thus provide a barrier of complexity between the paintings and their exterior. By contrast, self-similarity and anisotropy values of images of framed paintings were intermediate between the images of the paintings and the museum scenes, i.e., the frames provided a transition between the paintings and their surround. We also observed differences between the three museums that may reflect modified frame usage in different art periods. For example, frames in the museum for 20th century art tended to be smaller and less complex than in the two other two museums that exhibit paintings from earlier art periods (13th–18th century and 19th century, respectively). Finally, we found that the three properties did not depend on the type of reproduction of the paintings (photographs in museums, scans from books or images from the Google Art Project). To the best of our knowledge, this study is the first to investigate the relation between frames and paintings by measuring physically defined, higher-order image properties.

## Introduction

In his essay on the picture frame, Simmel (1902) proposed that the function of the frame is to separate a work of art, which represents a world on its own and does not require any relation to the exterior, from its surrounds. The frame thereby helps to place the artwork at a distance to the exterior, from which the observer can aesthetically enjoy the picture. Moreover, according to Simmel, the qualities of the picture frame assist and give meaning to the inner unity of the picture. Therefore, the frame has a dual function: the outer boundaries defend the picture against the exterior and the inner boundaries support a unifying integration with respect to the picture.

Ortega and Gasset (1921) emphasized the mutual dependence between the frame and the painting. Without the frame, the contents of the painting “seem to spill out over the four sides of the canvas” and “the frame constantly demands a picture with which to fill its interior.” In his view, the frame is a neutral object that isolates the imaginary island of the artwork from the surrounding reality on all sides. Indeed, the demarcation of the inner aspects of an artwork from its surrounding (external) reality is central to the definition of an object as an artwork also in a more recent philosophical theory of art (Danto, 2003). Wiesing (2006) suggested that the visible frame around a picture helps the observer to realize that the picture—even if it depicts an object realistically—is unreal and does not take part in the reality surrounding it. Interestingly, when reproduced in books or displayed electronically (e.g., in the Google Art Project), images of artworks are generally shown without frames, perhaps because it is more obvious that they are unreal. Since Simmel's and Ortega y Gasset's writings, novel forms of 2d visual art have emerged that do not require a frame for their presentation. Nevertheless, to this date, most paintings in traditional art museums are shown with frames.

Two additional aspects of frames should be mentioned. First, not all frames fit all paintings. To select the right type of frame for a given painting can be subject to careful aesthetic deliberations (Mendgen, 1995; Mitchell and Roberts, 1996; Siefert, 2010). It must therefore be assumed that frames interact perceptually with artworks, as Ortega y Gasset suggested (see above). The structure of a frame has specific properties that may relate to those of the artwork. Second, like the artworks themselves, the visual appearance of frames can change from one art period to another (Mitchell and Roberts, 1996; Siefert, 2010). For example, paintings of the Baroque or Renaissance period are often presented with complex frames while modern paintings of the 20th century are framed with simpler frames in general. However, beyond cultural factors, there may also be rules for framing that are similar across art periods and cultures. Such rules may possibly originate from perceptual mechanisms that are universal amongst humans, as has been proposed for artworks (Zeki, 1999; Redies, 2007).

Systematic studies that have investigated the physical properties of frames by objective and reproducible means are rare. In contrast, there are many studies on how visual objects must be positioned within the boundaries of a picture to yield an aesthetically pleasing result (for examples, see Arnheim, 1982; Palmer et al., 2008; McManus et al., 2011a,b). Overall, these studies provide evidence for reproducible and reliable aesthetic preferences that await explanation by a theory of visual aesthetic composition (McManus et al., 2011a). Also, there are many practical instructions for framing and art historical studies on framing preferences (Mitchell and Roberts, 1996).

In experimental aesthetics, it has been demonstrated that large subsets of visually pleasing images, including graphic visual artworks of Western and East Asian provenance, have specific global image properties (Graham and Field, 2007; Redies et al., 2007b; Graham and Redies, 2010). For example, in the Fourier domain, radially averaged (1d) power that is plotted as a function of spatial frequency, falls off according to a power law with a slope of around −2 in log-log plots (1/f^2^ characteristics). Power spectra with this slope value are scale-invariant, i.e., they do not change as one zooms in and out of the images, which are self-similar in the spatial domain. Interestingly, artworks share this property with complex natural scenes (Burton and Moorhead, 1987; Field, 1987; Tolhurst et al., 1992). Some other types of images that are produced manually by humans, such as handwritten text, do not exhibit this property (Melmer et al., 2013). It has been proposed that some artists create artworks by adapting them to the sensory coding in the human visual system (Zeki, 1999; Redies, 2007; Redies et al., 2007a), which itself is adapted to process the statistics of natural scenes.

More recently, self-similarity in large subsets of artworks has been determined with the Pyramid of Histograms of Oriented Gradients (PHOG) method (Amirshahi et al., 2012; Redies et al., 2012), a computational technique that was developed originally for object recognition and image categorization in digital image processing (Bosch et al., 2007). This method calculates the strengths of oriented luminance gradients in an image and plots them as a function of gradient orientation. With this method, self-similarity and two other properties previously studied in the context of aesthetic perception, complexity (Berlyne, 1971; Forsythe et al., 2011) and anisotropy (Koch et al., 2010; Melmer et al., 2013), were measured in a wide variety of images of diverse subject matters (man-made and natural) with different levels of aesthetic claim.

**Figure 1:**
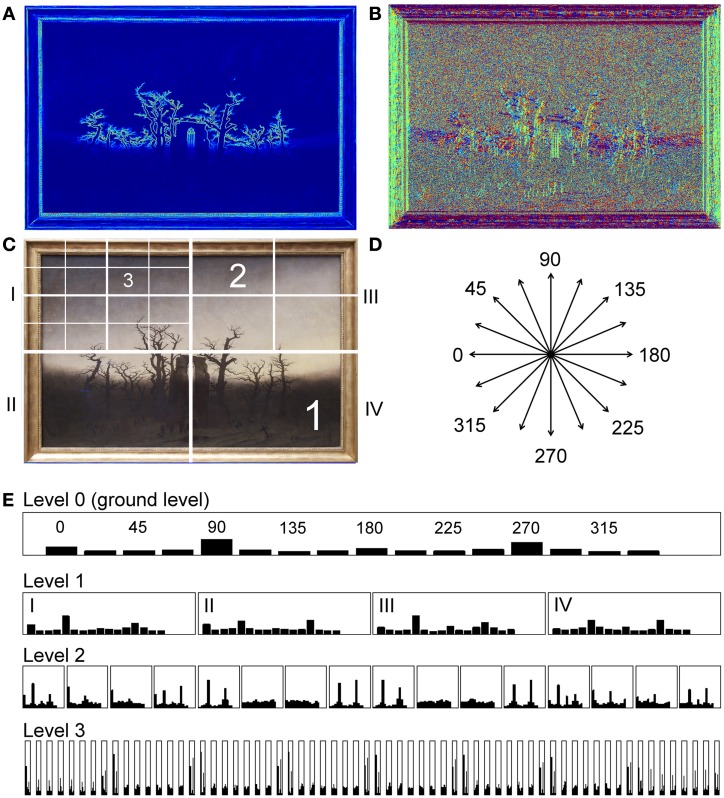
**Illustration of the image analysis. (A)** Image of gradient strength for the painting shown in Figure 3B. Gradient strength is represented by rainbow pseudo-color coding. **(B)** Image of gradient orientation represented by different colors (e.g., red for horizontal orientations and green for vertical orientations). **(C)** Diagram of the section sizes at the different levels (1–3) of the PHOG analysis. There are four sections at level 1, 16 sections at level 2, and 64 sections at level 3. Not all sections are shown for levels 2 and 3. **(D)** Orientations of the 16 bins that are used for calculating the HOG features. **(E)** HOG features for the ground level (level 0, top histogram) and for levels 1–3. The Arabic numerals indicate the binned orientations [see **(D)**]. The Roman numerals indicate the four sections at level 1 [compare to **(C)**]. The painting was reproduced with kind permission from © Staatliche Museen zu Berlin, Nationalgalerie.

**Figure 2:**
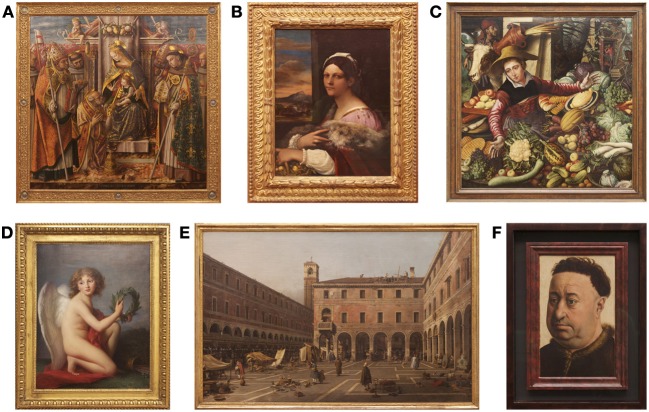
**Examples of the framed paintings *(PwF images)* analyzed**. All paintings are from the Painting Gallery [**(A)** Carlo Crivelli, 1488/89; **(B)** Sebastiano Luciani, 1513; **(C)** Pieter Aertsen, 1567; **(D)** Élisabeth-Louise Vigée-Lebrun, 1789; **(E)** Canaletto, 1758/59; **(F)** Master of Flémalle, about 1430/35]. The measures calculated for these images are provided in Table 1. Reproduced with kind permission from © Gemäldegalerie, Staatliche Museen zu Berlin—Preußischer Kulturbesitz, Eigentum des Kaiser Friedrich-Museums-Vereins **(A–D,F)**, and Leihgabe der Streitschen Stiftung, Berlin **(E)**.

Figure 1 gives an example of the image calculations (for more details, see Section Image analysis). Briefly, for the framed painting in Figure 1C, the image of gradient strength is shown with pseudo-color coding in Figure 1A, and the image of the gradient orientations in Figure 1B. For 16 bins of orientations (Figure 1D), the strength of the oriented gradients is calculated, resulting in the histogram of the gradient strengths for all orientation bins in Figure 1E (top histogram). The sum of the gradients strength across all orientations serves as a measure of complexity. The measure of self-similarity indicates how self-similar the histograms at different levels of spatial analysis (layers 1–3; see schematic diagram in Figure 1C) are to the ground level (level 0) histogram. Finally, anisotropy is a measure for how different the overall strength of gradients is across orientations. Low anisotropy values indicate that all orientations are represented at about equal strength. If one or a few orientations in an image are more prominent than others, the anisotropy value is higher. Table 1 lists some of these values that were calculated for the exemplary images of framed paintings displayed in Figure 2.

**Table 1:** **Results for the paintings with frames *(PwF images)* shown in Figure 2**.

**Panel in Figure 2**	**Artist (year)**	**Image size [sqm]**	**Frame area [%]**	**Complexity of**		**Specific complexity of frame**	**Self- similarity at level 3^d^**	**Aniso-tropy^d^**
				**Painting^b^**	**Frame^c^**			
A	Carlo Crivelli (1488/89)	3.74	26.1	9.53	4.62	17.7	0.73	0.0021
B	Sebastiano Luciani (1513)	0.48	50.6	3.03	6.85	13.5	0.49	0.0034
C	Pieter Aertsen (1567)	1.22	27.4	8.59	2.92	10.7	0.68	0.0047
D	E. Vigée-Lebrun (1789)	0.87	44.1	2.38	6.02	13.7	0.38	0.0009
E	Canaletto (1758/59)	2.21	13.8	6.65	2.11	15.2	0.58	0.0078
F	Master of Flémalle (about 1430/35)	0.05	65.5	2.74	2.17	3.3	0.59	0.0048
Median^a^		0.71	36.7	4.24	3.74	10.4	0.52	0.0052
Mean^a^		1.25	38.9	4.50	3.98	10.5	0.52	0.0056
*S.D.*		±1.25	±11.3	±1.44	±1.35	±3.1	±0.08	±0.0025

a for all paintings from the Painting Gallery (n = 108).

b for painting without frame (P images).

c for an image of the frame with a gray central area.

d calculated for images containing paintings and their frames (PwF images) at level 1.

Redies et al. (2012) showed that large subsets of visual artworks of Western provenance are characterized by a specific combination of these three measures (high self-similarity, intermediate complexity and low anisotropy) that distinguish them from a wide variety of image categories with no or lesser artistic claim.

**Figure 3:**
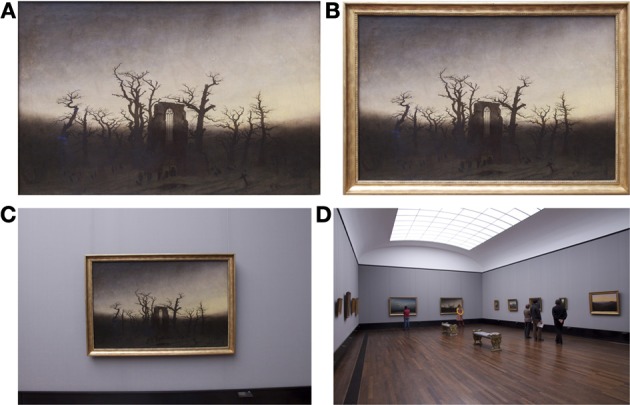
**Image categories analyzed**. Images of a painting without frame [*P image*; **(A)**], of the painting with its frame [*PwF image*; **(B)**], of a scene containing the painting, its frame and the immediate surround [*PwF/S image*; **(C)**], and a museum scene [*MSc image*; **(D)**] are shown. The painting by Caspar David Friedrich is entitled “Abbey Among Oak Trees” (1809/10) and was reproduced with kind permission from © Staatliche Museen zu Berlin, Nationalgalerie.

In the present work, we use the same method to study statistical image properties of framed paintings in museums. Specifically, we asked the following questions:

1. How do the image properties change as one zooms out from the paintings to their frames, their immediate surround and the interior of the museums (Figure 3)?
2. Can any systematic relations be found between the image properties of paintings and their frames?
3. Do museums that exhibit different art periods diverge in their frame usage?
4. Do the image properties vary between different representations of paintings (for example, in art books and in the internet)?

To answer these questions, we analyzed images of paintings from three major art museums that were chosen because they covered different periods of Western art: the Gemäldegalerie in Berlin (Painting Gallery; Old Master Paintings, 13th to 18th century), the Alte Nationalgalerie in Berlin (Old National Gallery; mostly 19th century paintings), and the Kunstsammlung Nordrhein-Westfalen in Düsseldorf (Art Collection of the State of North Rhine-Westfalia; 20th century paintings). A comparison of the three museums allowed us to assess which frame-related properties are stable across Western art periods and which depend on particular styles of art. To answer question (4), we analyzed photographs taken in the museums, images of respective paintings that were scanned from art books, and images downloaded from a web-based image depository (Google Art Project).

## Materials and methods

### Image data

**Figure 4:**
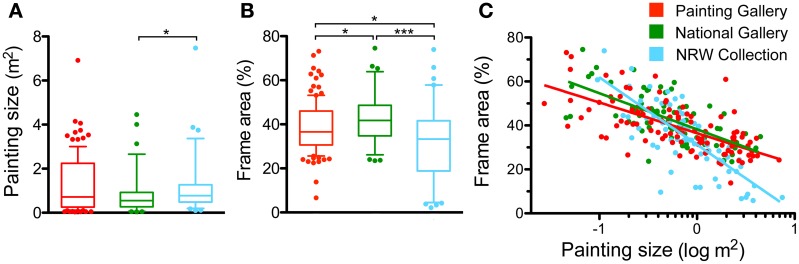
**Means of painting size and relative frame size**. The physical size of the paintings (without frame) is given in m^2^ **(A)** and the relative frame size as the percentage of the area that each frame covers in the *PwF image* **(B)**. The whiskers represent the 5–95 percentiles. Differences are significant at *p* < 0.05 (^*^) and *p* < 0.001 (^***^), as indicated. **(C)** Scatter graph of relative frame size plotted as a function of painting size. Each dot represents one painting. The lines represent the results of linear regression analysis (*p* < 0.001). Spearman's correlation coefficients are: Painting Gallery, *r*: −0.69; National Gallery, *r*: −0.68; and NRW Collection, *r*: −0.76. Colors indicate the different museums, as indicated in **(C)**.

For analysis, six datasets of images were generated: (1) Photographs of paintings without frames *(P images;* Figure 3A), (2) photographs of paintings with frames *(PwF images*; Figure 3B), (3) photographs of scenes that included paintings, their frames and the immediate surround *(PwF/S images*; Figure 3C), and (4) photographs of entire museum scenes *(MSc images*; Figure 3D). Note that all images contained paintings, but differed in how much their surround was included in the scene. From (1) to (4), the observer's view zooms out as the viewing distance increases. A similar approach has been taken to analyze images of natural and man-made scenes previously (Torralba and Oliva, 2003; Redies et al., 2012; Braun et al., 2013). The segmentation of the scenes in (1)–(3) coincides with the borders of the frames. In addition, we analyzed (5) *P images* that were scanned from art books, and (6) *P images* that were downloaded from a web-based image depository (Google Art Project). An effort was made to include, as much as possible, the same paintings in all datasets so that different conditions of viewing or reproduction could be compared directly for the same paintings. Because not all paintings were hanging in the museums at the time of photography, we included only those paintings in the analysis that we were available to us both for photography and in the catalogs. This selection favored the inclusion of all major art styles and a large variety of artists from each museum. Table 2 lists the number of artists and images analyzed and the art periods covered by the three museums.

**Table 2:** **Description of museums, number of artists and number of images analyzed**.

**Museum**	**Major art periods**	**Century**	**No. of artists**	**Number of images analyzed**					
				***P images*^d^ (photo-graphs)**	***PwF images*^e^**	***PwF/S images*^f^**	***MSc images*^g^**	***P images* (scans)**	***P images* (down-loads^h^)**
Painting Gallery^a^ (Old Master paintings)	Gothic, Renaissance, Manierism, Baroque, Classicism, etc.	13th–18th	94	120	120	117	89	27	27
National Gallery^b^	Realism, Romanticism, Impressionism, etc.	19th	30	75	75	77	39	31	31
NRW Collection^c^	Expressionism, Surrealism, Cubism, abstract art, etc.	20th	42	85	85	87	35	–	–

a Gemäldegalerie–Stiftung Preussischer Kulturbesitz, Berlin.

b Alte Nationalgalerie–Stiftung Preussischer Kulturbesitz, Berlin.

c Sammlung Nordrhein-Westfalen (NRW), Düsseldorf.

d Images that contain a painting only.

e Images that contain a painting and its frame.

f Images that contain a painting, its frames and the immediate surround.

g Images of museum scenes.

h From the Wikimedia Commons website (Google Art Project, see Methods).

#### Photographs of museum paintings and scenes

Photographs were taken in RAW format with a 15.1 megapixel digital camera (EOS 500D with EF-S15-85 mm f/3.5-5.6 IS USM lens; Canon, Tokyo, Japan) by one of the authors (Christoph Redies). The camera was mounted on a tripod so that the objective lens was at about 1.6 m height above the floor level. The camera was set to an aperture of F9.0 and ISO 400. White balance was adjusted to artificial light (about 4000 K) because this corresponded to the museum lighting in general.

Rectangular paintings were photographed only because we were unable to analyze non-rectangular images with our computer programs (see Section Image analysis). Each painting was photographed so that the painting and the frame filled most of the photograph. Following photography, lens distortion was corrected with the lens correction facility of the Photoshop program (CS2, Adobe, Mountain View, CA) for each image separately. Digital images were then carefully cropped to obtain rectangular images of the complete paintings without frame *(P images)* with the Photoshop program (for an example, see Figure 3A). In a second cropping session, we generated rectangular images of the paintings with their frames (*PwF images*; Figure 3B). Paintings with frames that had irregular inner boundaries (less than about 10% of paintings photographed) were excluded from the analysis because it was not possible to crop the photographs to obtain images containing the entire painting but without parts of the frame. Images of paintings with irregular outer frame boundaries were cropped so that the frames were included completely in the images. This was considered less critical because the surrounding area generally had a uniform structure.

Next, we obtained images of framed paintings with their surround (*PwF/S images*). These images were cropped so that the width of the resulting image measured about twice the width of the paintings (Figure 3C). Some of these images (39% in the Painting Gallery, 42% in the National Gallery, and 25% in the NRW Collection) contained not only the uniform wall behind the paintings but also visual features of moderate to high contrast, for example, parts of the ceiling, the floor or other paintings.

In each museum, photographs of exhibition rooms or corridors were taken (here called museum scenes or *MSc images*), usually with several paintings and sometimes with a few visitors in the picture (Figure 3D). In the Painting Gallery (PG), visitors were included in 14 out of 89 *MSc images*, in the National Gallery (NG) in 29 out of 39 images, and in the NRW Collection 2 out of 35 images (average of 2.1 persons/image with persons). The authors did not have permission to ask visitors to leave the exhibition rooms where they took photographs. An effort was made to keep the number of photographed visitors as low as possible and to avoid close-up views of visitors in the *MSc images*. The *MSc images* were not cropped. There was no difference in the calculated values (Sections Self-similarity and Anisotropy) between *MSc images* with visitors and without visitors for the PG and the NG (two-tailed Mann-Whitney test).

#### Scans of images from museum catalogs

*P images* were also obtained by scanning exhibition catalogs [PG (Berlin, 2010), NG (Maaz, 1999; Keisch, 2005; Wesenberg, 2011), and NRW Collection (Essers et al., 2000)]. Care was taken to select plates of high quality and of large size. Scanning was carried out with calibrated scanners (Perfection 3200 Photo and Perfection V700 Photo, Epson, Nagano, Japan) at a high resolution in 24-bit color scale.

#### Images from the google art project

Finally, *P images* were obtained from the Google Art Project, an online platform through which the public can access high-resolution images of artworks (www.googleartproject.com). For the PG and the NG, all paintings that had been scanned from art books were downloaded if they were available in the platform. There were no images from the NRW Collection. The image files of the paintings were accessed through the Wikimedia Commons website (commons.wikimedia.org). The size of the downloaded image files was 2–36 Mb. We were not able to obtain technical information on how the images were acquired.

### Image analysis

Besides some general measurements like frame and painting size (see Section General image measures), we calculated values for self-similarity, complexity and anisotropy for each image. These values have been previously studied in visually pleasing images (see Introduction) and were obtained with the PHOG method (Dalal and Triggs, 2005; Bosch et al., 2007). The PHOG method was originally developed for image categorization and object recognition.

#### General image measures

The size of each painting was calculated on the basis of the dimensions that were given in the exhibition catalogs and expressed as the area occupied by the painting in m^2^. The frame area was defined as the area, which the frame covered in the *PwF images*, expressed in percent of the total *PwF image* area.

#### Image resizing

Because self-similarity, complexity and anisotropy depend on image size (Figure 7), images were reduced to a uniform size to 1 million pixels by isotropic scaling, unless stated otherwise. For the comparison of photographs, scans and online versions of the paintings from the Google Art Project (Figure 12), the image size was decreased to 100,000 pixels by bicubic interpolation. This reduction was required in order to eliminate halftone dots from the image that were visible at a resolution of 1 million pixels in some of the scanned images.

#### Calculation of gradient images

For the PHOG analysis, images of oriented gradients were calculated, as described before (Amirshahi et al., 2012; Redies et al., 2012; Braun et al., 2013). The method is illustrated in Figure 1. In brief, color images were transformed into the Lab color space and the intensity of oriented gradients was calculated for each channel separately. A gradient image was then calculated based on the maximum gradients in either the L, a or b color channel, whichever was highest (Dalal and Triggs, 2005; Braun et al., 2013). For the framed painting displayed in Figure 3B, the strength and the orientation of the gradients are shown in Figures 1A,B, respectively, for each pixel of the image. This gradient image was used to obtain the three measures described below.

#### Self-similarity

Self-similarity was calculated with a metric that was derived from the PHOG descriptor (Bosch et al., 2007). For the descriptor, histograms of oriented luminance gradients [HOG features (Dalal and Triggs, 2005)] were obtained for each section at different levels of an image pyramid (Figure 1C; Amirshahi et al., 2012; Braun et al., 2013). To calculate the HOG feature for each section, the gradient orientations were separated into 16 bins of equal size across 360° (Figure 1D) and the strength of the orientations in each bin was measured. At the basis of this pyramid (i.e., the ground level or level 0), the HOG feature for the entire image was calculated (top histogram in Figure 1E). The image was then divided into four equally sized rectangles to yield level 1 of the pyramid. Again, for each subsection at this level, the HOG features were calculated and each subsection was then again divided into four equally sized rectangles to generate the next level of the pyramid. Level 2 thus contained 16 sections, level 3 contained 64 sections, and level 4 contained 256 sections (Figure 1C). For each section at a given level, the HOG feature was calculated (Figure 1E). In each individual histogram, the values were normalized so that their sum was one.

To obtain a measure for self-similarity, the HOG features at different levels of the pyramid were compared with the ground level histogram. The similarity of histograms was determined by the Histogram Intersection Kernel function (Barla et al., 2002). As described in the Results section, obtained values differed depending on the level analyzed (Figure 8). For this reason, we calculated self-similarity values uniformly until level 3 of the pyramid. Level 3 was chosen because it gave distinct results for the different image categories. Higher levels yielded sections that were exceedingly small and had increasingly uniform luminance distributions with fewer gradients, producing more unstable results (Amirshahi et al., 2012).

#### Complexity

Physical complexity of the images was defined as the mean value of gradient strength over the gradient image at the ground level across all orientations (Dalal and Triggs, 2005; Braun et al., 2013). This complexity measure is similar to edge density in that both represent gradient-based features. The exact relation to other measures of physical image complexity (Boon et al., 2011; Forsythe et al., 2011) is unclear at present. We did not measure subjective complexity perceived by observers, which may be deviate from physical complexity (Forsythe et al., 2011).

#### Anisotropy

The variance of the luminance gradient strengths across the 16 orientation bins was calculated as a measure of anisotropy (Braun et al., 2013). This measure indicates how much the strength of the gradients differed across orientations. If not otherwise stated, the calculation was carried out at level 1.

#### Statistical analysis

Differences between image categories were statistically evaluated by a non-parametric one-way analysis of variance test (Kruskal-Wallis test with Dunn's multiple comparison post-test). To indicate the standardized difference between two means, Cohen's *d*-value was calculated.

## Results

### Overview of images analyzed

We studied framed paintings from three German art museums that cover major periods of Western art. Table 2 lists the art periods, the number of artists and the number of images analyzed for each museum. Figure 2 shows six framed paintings from the NG as examples.

**Figure 5:**
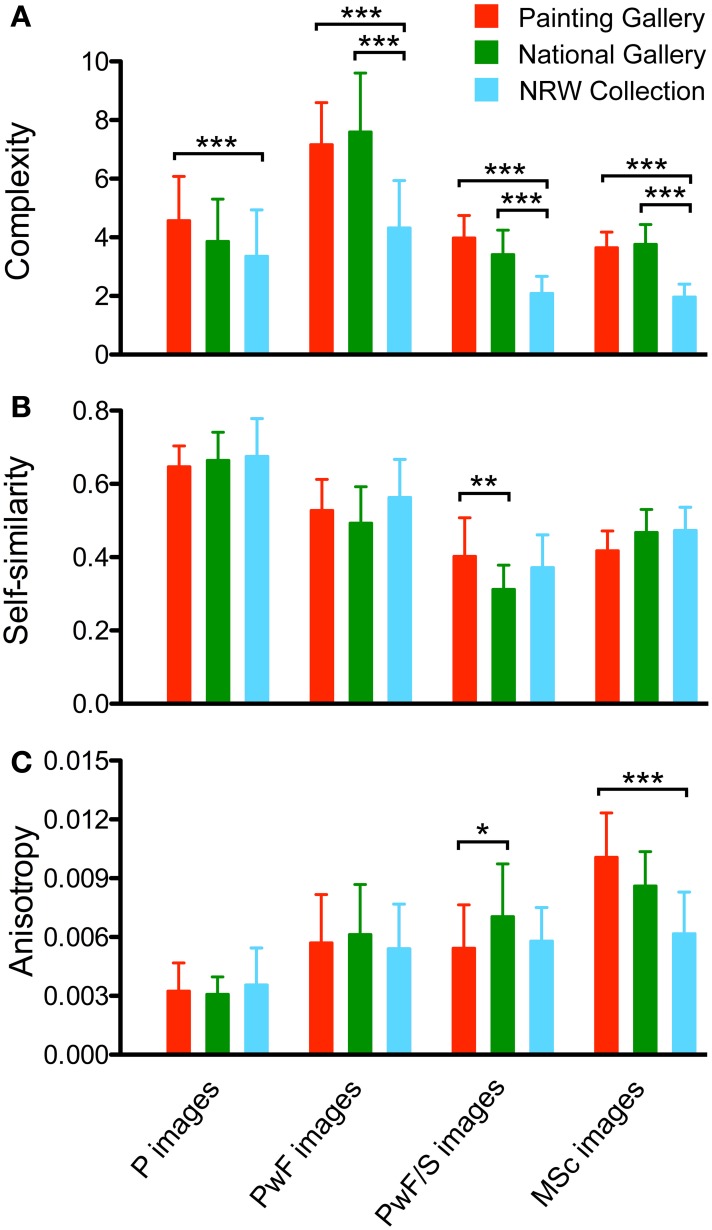
**Results for complexity (A) self-similarity (B), and anisotropy (C)**. The four image categories analyzed are indicated at the bottom of the figure for all panels. Values represent the mean ± 1 *SD*. Colors indicate the different museums, as indicated in **(A)**. Differences are significant at *p* < 0.05 (^*^), *p* < 0.01 (^**^) and *p* < 0.001 (^***^), as shown. Significance levels are shown for comparisons within image categories only.

Figures 4A,B represents box diagrams of the physical size of the paintings and the relative size of the frames, expressed as percentage of the area, which the frames occupy in images of paintings and their frames (*PwF images*; see Section Results for Different Types of Scenes with Paintings). In Figures 4, 5 and 9–12, the following color code is used: red, Painting Gallery (PG); green, National Gallery (NG); and light blue, NRW collection (NRW).

Painting size in the three museums differed slightly only between the NG (mean 0.84 m^2^) and the NRW Collection (1.15 m^2^; *p* < 0.05, Cohen's *d:* 0.31). The frame area percentage was lower for the NRW Collection (31.1%) than for the NG dataset (43.0%; *p* < 0.001, *d:* 0.78) and for the PG dataset (38.5%; *p* < 0.05, *d:* 0.54; Figure 4B). Because of this fundamental difference in relative frame size, we carried out the rest of the analysis for each museum separately, with few exceptions.

In all three museums, painting size correlated negatively with the frame area percentage (*p* < 0.001). In other words, larger paintings had relatively smaller frames. Figure 4C shows scatter diagrams for the two measures with painting size plotted on a logarithmic scale. The lines represent best fits in a linear regression analysis.

### Results for different types of scenes with paintings

We next asked whether there are differences in image statistics between the following image categories: (1) images of paintings without frames (*P images*; for an example, see Figure 3A), (2) images that contain the same paintings with their frames (*PwF images*; Figure 3B), and (3) the framed paintings with their immediate surround (*PwF/S images*; Figure 3C). The surround was defined as the rectangular area around the painting that had about twice the width of the painting. In addition, (4) photographs of large-vista views across exhibition halls and museum corridors were included in the analysis (*MSc images*; Figure 3D). This sequence of image categories corresponds to a zooming out of the observer's view or increasing the distance for viewing the paintings.

Figure 5 shows results for complexity (Figure 5A), self-similarity (Figure 5B) and anisotropy (Figure 5C) for each of the three museums. In this figure, results of significance testing for differences within image categories (see Section Self-similarity) are indicated. For differences between image categories, see the text below. For complexity and self-similarity, a summary of all *p*-levels is provided in Table 3.

**Table 3:** **Significance levels (*p* < …) for the differences in complexity (upper right half of the table) and self-similarity (lower left half) between the different image categories in the three museums (Kruskal-Wallis test with Dunn's post-test)**.

	**Painting Gallery**				**National Gallery**				**NRW Collection**			
	***P***	***PwF***	***PwF/S***	***MSc***	***P***	***PwF***	***PwF/S***	***MSc***	***P***	***PwF***	***PwF/S***	***MSc***
Painting Gallery												
*P*	–	0.001	ns	0.05	ns	0.001	0.001	ns	0.001	ns	0.001	0.001
*PwF*	0.001	–	0.001	0.001	0.001	ns	0.001	0.001	0.001	0.001	0.001	0.001
*PwF/S*	0.001	0.001	–	ns	ns	0.001	ns	ns	0.01	ns	0.001	0.001
*MSc*	0.001	0.001	ns	–	ns	0.001	ns	ns	ns	ns	0.001	0.001
National Gallery												
*P*	ns	0.001		0.001	–	0.001	ns	ns	ns	ns	0.001	0.001
*PwF*	0.001	ns	0.001	0.05	0.001	–	0.001	0.001	0.001	0.001	0.001	0.001
*PwF/S*	0.001	0.001	0.01	0.001	0.001	0.001	–	ns	ns	ns	0.001	0.001
*MSc*	0.001	ns	ns		0.001	ns	0.001	–	ns	ns	0.001	0.001
NRW Collection												
*P*	ns	0.001	0.001	0.001	ns	0.001	0.001	0.001	–	0.01	0.001	0.001
*PwF*	0.001	ns	0.001	0.001	0.001	ns	0.001	0.05	0.001	–	0.001	0.001
*PwF/S*	0.001	0.001	ns	ns	0.001	0.001	ns	0.05	0.001	0.001	–	ns
*MSc*	0.001	ns	ns	ns	0.001	ns	0.001	ns	0.001	ns	0.05	–

Abbreviations: MSc, images of museum scenes (MSc images); ns, not significant; P, images that contain a painting only (P images); PwF, images that contain a painting and its frame (PwF images); PwF/S, images that contain a painting, its frames and the immediate surround (PwF/S images).

The *complexity* of the *P images* (Figure 5A) was lower than that of the *PwF images* in all three museums (*p* < 0.001, *d*: 1.76 for PG; *p* < 0.001, *d*: 2.15 for NG; and *p* < 0.01, *d*: 0.61 for NRW). Complexity was lower for the *PwF/S images* than for the *PwF images* (all *p* < 0.001; *d*: 2.77 for PG, 2.76 for NG, and 1.85 for NRW). This result was expected because most of the paintings' surround consists of uniformly structured wallpaper. There was no significant difference in complexity between the *P images* and the *PwF/S images*, except for the NRW Collection (*p* < 0.001, *d*: 1.18). *MSc images* did not differ in their complexity from *PwF/S images*. For all image categories, complexity was significantly lower for the NRW collection than for the other two museums (*p* < 0.001), except for the NRW/NG comparison of unframed paintings.

The average *self-similarity* of the paintings (Figure 5B) is higher for the *P images* than for the other image categories (*p* < 0.001; *d*: 1.66 to 4.13 for PG, 1.95 to 4.97 for NG, and 1.09 to 3.16 for NRW). For all museums, *PwF images* show higher self-similarity values than *PwF/S images* (all *p* < 0.001; *d*: 1.32 for PG, 2.16 for NG, and 2.00 for NRW). For the PG only, values for *PwF images* are higher than those for *MSc images* (*p* < 0.001, *d*: 1.51). *PwF/S images* have lower self-similarity values than *MSc images* in the NG (*p* < 0.001, *d:* 2.41) and in the NRW Collection (*p* < 0.05, *d:* 1.24). Within the image categories, self-similarity is not significantly different (except for the PG/NG comparison of *PwF/S images*, *p* < 0.01, *d:* 0.98).

*Anisotropy* (Figure 5C) was calculated at level 1 and is lower for the *P images* than for any of the other image categories in all three museums (*p* < 0.001, *d:* 1.18 to 3.75 for PG, 1.61 to 4.48 for NG, and 0.89 to 1.34 for NRW). The increase seen for *PwF images* is expected because the borders of the frames represent mostly cardinal (vertical and horizontal) orientations. Also expected is the lack of a difference between *PwF images* and *PwF/S images* because the surrounding wallpaper has few orientation cues in any direction in general, although some *PwF/S images* show parts of the floor and ceiling.

**Figure 6:**
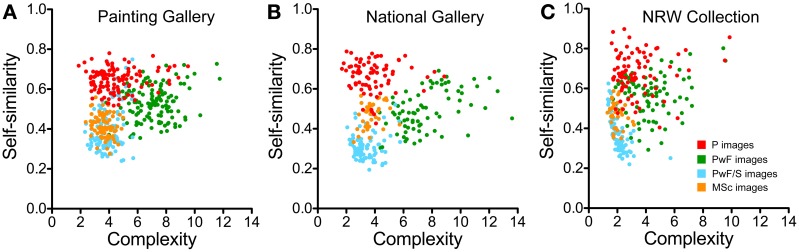
**Scatter graphs of self-similarity plotted as a function of image complexity**. Each dot represents one painting from the Painting Gallery **(A)**, the National Gallery **(B)**, and the NRW Collection **(C)**. Colors indicate the different image categories, as indicated in **(C)**.

Scatter plots for the three museums are displayed in Figure 6. Each dot represents the result for one image. The dots for the different image categories form distinct clusters that overlap partially. The overall position of the clusters is similar for all three museums. There were significant correlations between complexity and self-similarity for the following comparisons: *PwF/S images* in the PG (*p* < 0.05, Spearman *r:* 0.20) and in the NRW collection (*p* < 0.001, *r:* −0.79), and *PwF images* (*p* < 0.001, *r:* 0.48) and *MSc images* (*p* < 0.05, *r:* 0.41) in the NG.

In summary, when zooming out of images with paintings (*P images*), complexity initially increases as the frame is included in the images (*PwF images*), and then decreases again as more of the surround is contained in the images (*PwF/S images* and *MSc images*). This results is the same for all three museums. Self-similarity is higher for *P images* than for any of the other image categories. Anisotropy is lowest for *P images* and increases as more and more surrounding structures become visible in the images, due to the predominance of cardinal orientations in the frames and the museum architecture.

### Dependence of measured values on image size and the PHOG level

**Figure 7:**
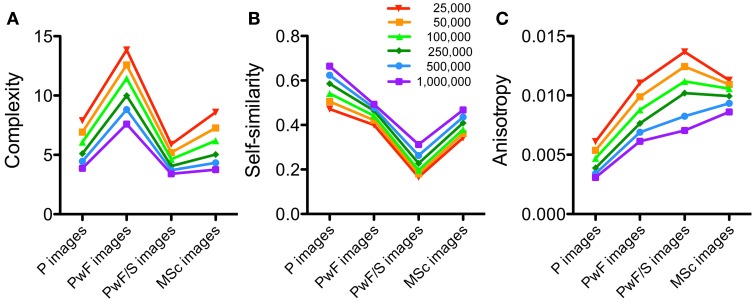
**Results for different image resolutions**. The National Gallery dataset was analyzed for image complexity **(A)**, self-similarity **(B)**, and anisotropy **(C)**. Image categories are indicated below the x axis. Colors indicate the different image resolutions, expressed in number of pixels, as indicated in **(B)**.

In the present study, all images were normalized to a uniform image size of 1,000,000 pixels (as the product of width x height) because preliminary experiments had shown that the statistical measures depend on image size. This dependence was studied in more detail by calculating the measures again for images normalized to sizes ranging from 25,000 to 1,000,000 pixels. As an example, the NG dataset was analyzed. Results in Figure 7 confirm that the general tendencies described above are similar for all three measures and for all resolutions studied, although absolute values differed.

**Figure 8:**
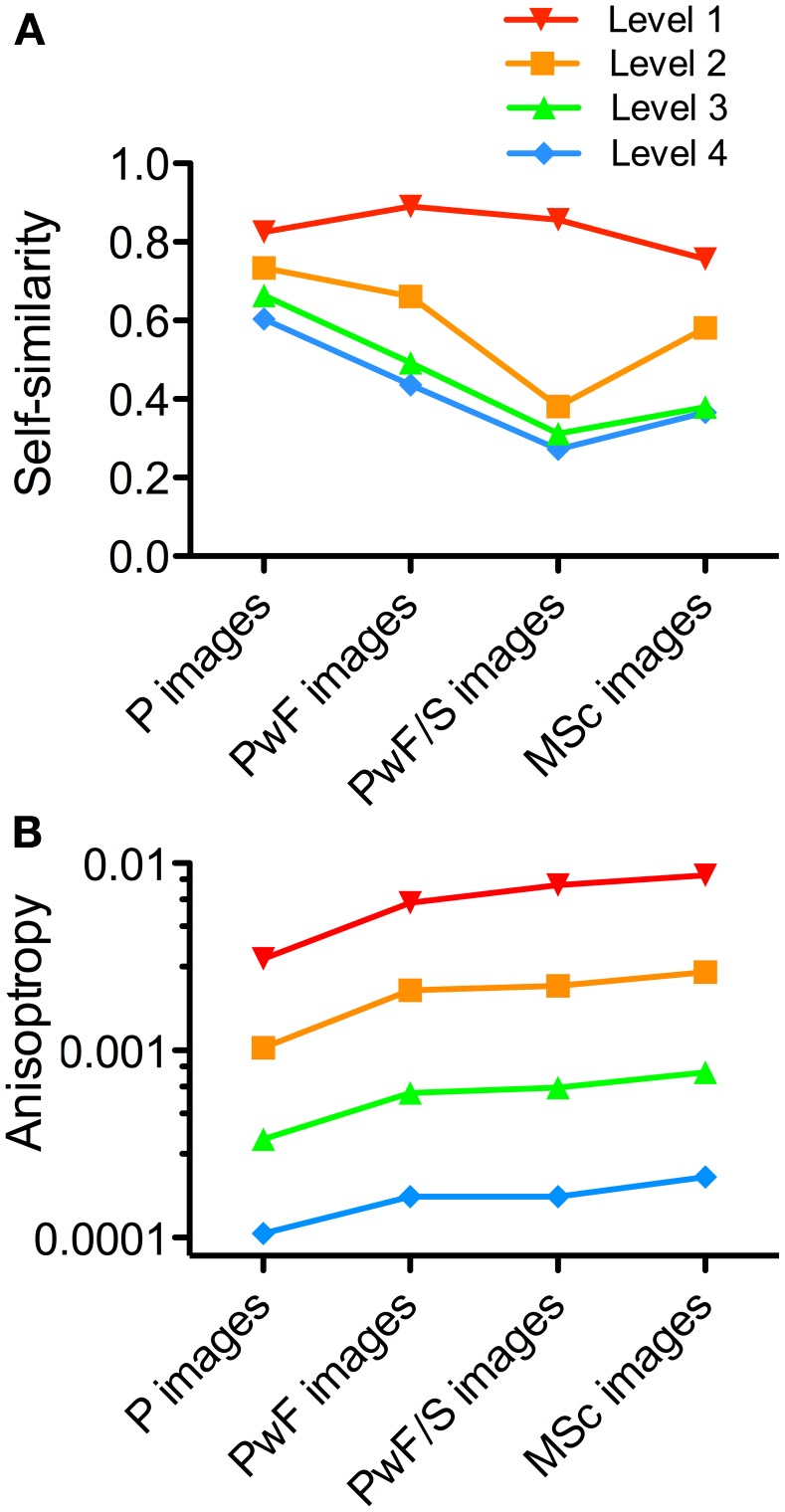
**Results for self-similarity **(A)** and anisotropy **(B)** at different levels of the PHOG analysis**. The National Gallery dataset was analyzed. Image categories are indicated below the x axis. Colors indicate the different PHOG levels of analysis, as indicated above the graph.

Moreover, for Figure 5, self-similarity and anisotropy were calculated at level 3 and level 1, respectively, of the PHOG pyramid. Because these measures depend on the level chosen for calculation (Amirshahi et al., 2012), we obtained the values also for other levels. Again, the NG dataset served as an example. Results for self-similarity (Figure 8A) show that, at level 1, self-similarity values are similar for all image categories. This result is expected because at this level, images are divided into four equally sized rectangles, each of which consists of roughly equal portions of the painting frame and surround. At higher levels, results are similar to those at level 3. For the *P images*, the self-similarity values are more similar at all levels of analysis than those of the other image categories. Anisotropy values show similar relative differences between the image categories although the absolute values differ between levels (Figure 8B).

### Measuring frame complexity

**Figure 9:**
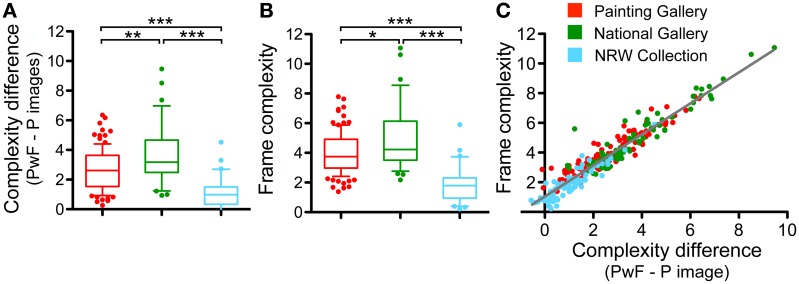
**Frame complexity**. Two different measures of frame complexity were obtained. First, the complexity difference between the painting with frame and without frame was calculated **(A)**. Second, complexity of each frame filled by a central area of uniform gray was calculated **(B)**. Colors represent the different museums, as indicated in **(C)**. The whiskers represent the 5–95 percentile. Differences between museums are significant at *p* < 0.05 (^*^), *p* < 0.01 (^**^), and *p* < 0.001 (^***^), as indicated. A scatter graph of the two measures is shown in **(C)**. Each dot represents one painting. The line represents the result of a linear regression analysis on all data points (*p* < 0.001, *r:* 0.94).

Of particular interest is the finding that the *PwF images* are more complex than the *P images* (Figure 5A). We therefore asked how complex the frames were in relation to the paintings at their interior. The complexity of a frame cannot be measured in isolation because the border between the frame and the painting contributes to the complexity of the framed paintings. We therefore measured frame complexity indirectly in two ways: First, we calculated the difference in complexity between *P images* and the *PwF images*. Results are shown in Figure 9A. Second, we replaced the area of the paintings in the *PwF images* by a homogeneous rectangle of uniform luminance and measured the complexity of these modified *PwF images*. Because the strength of the gradient calculated for the inner border of the frame depends on the contrast between the frame and the enclosed area, we compared the modified images with three luminance levels for the enclosed rectangle: 0% luminance (i.e., the painting was replaced by a black rectangle), 50% luminance (by a gray rectangle) or 100% luminance (by a white rectangle). Mean complexity values for the modified *PwF images* were 3.90 ± 1.87 *SD* for the black rectangle, 3.64 ± 1.91 *SD* for the gray rectangle, and 3.84 ± 1.94 *SD* for the white rectangle. The finding that the images with the gray rectangle had lowest complexity values was expected because most frames assume intermediate luminance levels. Consequently, their inner border shows less contrast with the gray rectangle than with the white or black one. Overall, the values for the three luminance levels differed only moderately. Figure 9C demonstrates that the values from the two measures of frame complexity correlate highly with each other (*p* < 0.001, *r:* 0.94), in support of the validity of both approaches.

For both measures, frame complexity values for the NRW collection were lower than for the other two museums (*p* < 0.001, *d:* 1.73 compared to PG, and 2.00 compared to NG), and lower for the PG than for the NG (*p* < 0.05, *d:* 0.60).

### Relation of frame properties and image properties

**Figure 10:**
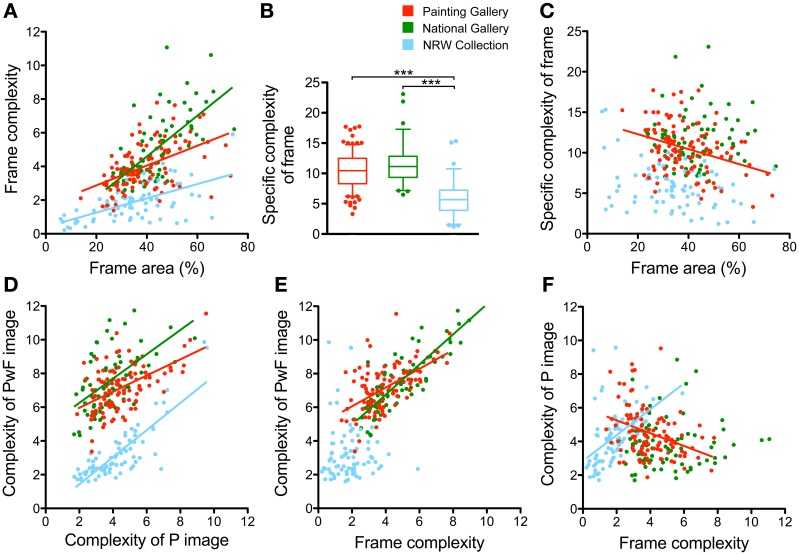
**Some relations between frame area, frame complexity and complexity of the paintings**. Scatter graphs **(A,C–F)** for different combinations of measures are shown (see labeling of the x axis and y axis). Each dot represents one painting. Colors represent the different museums, as indicated in **(B)**. The colored regression lines represent significant results of a linear regression analysis on all data points from one museum (same color coding as for the dots). Significance levels and Spearman r coefficients are mentioned in the text. **(B)** Box plot of the values for specific frame complexity. The whiskers represent the 5–95 percentile. Differences between museums are significant at *p* < 0.001 (^***^), as indicated.

**Figure 11:**
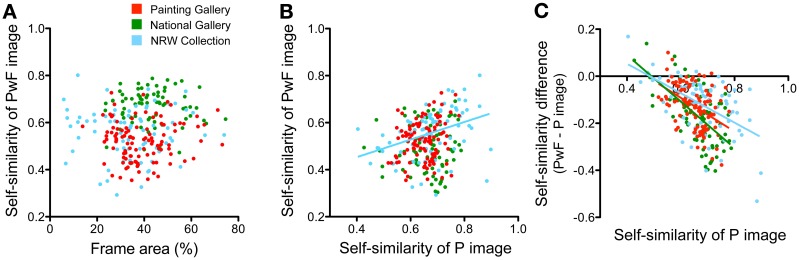
**Relations between frame area and self-similarity of paintings with or without frames**. Scatter graphs of different combinations of the measures (see labeling of the x axis and y axis) are shown **(A–C)**. Each dot represents one painting. Colors represent the different museums, as indicated in **(A)**. The colored regression lines represent significant results of a linear regression analysis on all data points from one museum (same color coding as for the dots). Significance levels and Spearman (*r*) coefficients are mentioned in the text.

Next, we asked whether the statistical image properties of the *P images* and the *PwF images* correlated with each other or with frame complexity. The calculation of complexity was based on *PwF images* with gray inner rectangles (see previous section). Results are displayed in Figures 10, 11. In the figures, fitted lines from linear regression analysis are shown for significant correlations only.

First, for all three museums, we found that frame complexity increases as the area that is occupied by the frame in the *PwF images* becomes larger (Figure 10A; *p* < 0.001, *r:* 0.51 for PG, 0.71 for NG, and 0.55 for NRW). In other words, the frame contributes more to the overall complexity of PwF images as the frames become wider, as we expected. But do wider frames also have a more complex structure? To answer this question, we measured the specific frame complexity, which was defined as the complexity of the frame divided by its relative area in the *PwF images*. Figure 10B shows that the specific complexity of the frames is smaller on average in the NRW Collection (5.83) than for the PG dataset (10.55; *p* < 0.001, *d:* 1.62) and the NG dataset (11.50; *p* < 0.001, *d:* 1.92). There was no overall correlation between specific frame complexity and frame area when all three museums were analyzed together (Figure 10C); only when the PG dataset was analyzed alone, a weak negative correlation was found (*p* < 0.01; Spearman *r*: −0.28). Also, the complexity of the *P images* tended to be lower for paintings with wider frames in the NG dataset (plots not shown; *p* < 0.01; *r*: −0.30), but not for the other two museums. In the NG dataset only, *PwF images* tended to be more complex when they had wider frames (plots not shown; *p* < 0.001; *r:* 0.45).

The complexity of the *PwF images* correlated moderately with the complexity of the *P images* for all three museums (Figure 10D; *p* < 0.001; *r:* 0.45 for PG; *r:* 0.51 for NG, *r:* 0.77 for NRW). It also correlated with frame complexity (Figure 10E; *p* < 0.001; *r:* 0.52 for PG; *r:* 0.82 for NG), except for the NRW Collection dataset. More complex *P images* tended to have more complex frames in the NRW Collection dataset (Figure 10F; *p* < 0.001, *r*: −0.38) and less complex ones in the PG dataset (Figure 10F; *p* < 0.001, *r:* 0.59). The correlation between the complexity of the *P images* and the specific complexity of the frame was weak for the PG (plots not shown; *p* < 0.05; *r*: −0.22) and the NRW Collection (plots not shown; *p* < 0.05; *r:* 0.28). However, the difference in specific complexity between paintings with and without frames was higher for less complex paintings for all three museums (plots not shown; *p* < 0.001, *r*: −0.58 for PG; *p* < 0.001, *r*: −0.40 for NG; *p* < 0.01, *r*: −0.36 for NRW).

There was no correlation between the frame area and the self-similarity of the *PwF images* (Figure 11A) or the *P images* (plots not shown). The self-similarity of *PwF images* tended to be higher for *P images* that were more self-similar, but only for the NRW Collection (Figure 11B; *p* < 0.001; *r:* 0.45). For all three museums, the difference in self-similarity between *P images* and *PwF images* was higher if *P images* were more self-similar (Figure 11C; *p* < 0.001; PG, *r*: −0.44; NG, *r*: −0.57; NRW, *r*: −0.48).

Anisotropy of the *P images* did not correlate with frame area. There was a weak correlation for anisotropy values between *P images* and *PwF images* for the PG dataset (plot not shown, *p* < 0001, *r:* 0.32) and the NG dataset (plot not shown, *p* < 0.05, *r:* 0.23) but not in the NRW Collection dataset.

### Comparison of different types of image reproduction

Finally, we asked whether *P images* that were generated by different reproduction techniques and for different presentational purposes vary in their statistical image properties. We compared reproductions of the same paintings obtained from the following sources: (1) Images photographed in the museums (see above), (2) images scanned from the museum catalogs, and (3) images downloaded from an electronic database (Google Art Project). We restricted this analysis to the paintings that were available in all three datasets. At the time of analysis, the Google Art Project database contained no images from the NRW Collection and only a limited number of the paintings that we had photographed and scanned for the PG (27 paintings) and NG (31 paintings).

**Figure 12:**
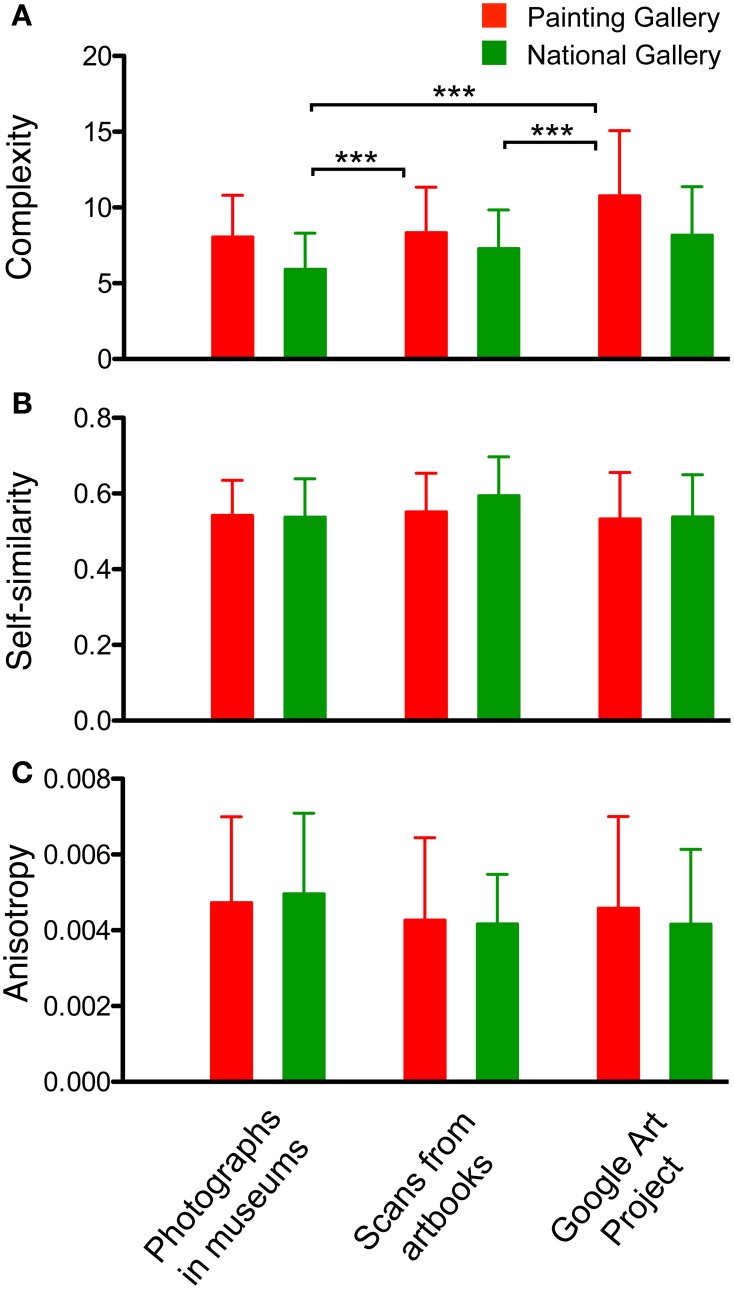
**Complexity (A), self-similarity (B) and anisotropy (C) of images of paintings (without frames)**. The three image categories analyzed were photographs of paintings, images of paintings scanned from art books and images of paintings downloaded from the Google Art Project, as indicated at the bottom for all panels. The same paintings from two museums (red, Painting Gallery; green, National Gallery) were analyzed. Values represent the mean ± *SD*. Differences are significant at *p* < 0.001 (^***^), as indicated.

Figure 12 shows that complexity, self-similarity and anisotropy are generally very similar for all types of painting representations. In particular, there was no significant difference for the comparison within museums. The significant difference in complexity between museums has been described above (Figure 5A). Because the analysis was carried out at a resolution of 100,000 pixels (see Methods), absolute values differed (Figure 7) from the preceding analysis, which was carried out at a resolution of 1 million pixels.

## Discussion

We studied the visual effects of frames on paintings that were photographed in three major art museums, each of which covered a different period of Western art. Three statistical measures (self-similarity, complexity and anisotropy) were calculated with a computer-based approach that is commonly used in digital image processing (Dalal and Triggs, 2005; Bosch et al., 2007). To the best of our knowledge, this is the first study to investigate statistical image properties of picture frames and their relation to paintings.

In the following sections, we will describe general characteristics of the frames that were common in all three museums. Second, we will discuss some methodological issues related to the statistical measures, as well as their significance and shortcomings. Third, we highlight differences in framing practice between the museums. Fourth, the measures are compared for different types of presentation of *P images*.

### General visual effects of frames

On the one hand, it is obvious that cultural aspects have an influence on the framing of paintings. For example, different types of frames have been used in different art periods (see Introduction). Some of these historic preferences are still apparent in museum exhibitions today. On the other hand, it may be asked whether frames have general effects on the visual appearance of paintings across major art periods. In the present study, we observed both types of phenomena in the three museums, which each covered a different art period (Table 2). While some of the common features are more or less trivial, others are novel and were not predicted by us. For example, it is perhaps not so surprising that the relative frame size of larger paintings is smaller on average than that of smaller paintings (Figure 4C). Also, it seems trivial that the complexity of *PwF images* correlates with both the complexity of the *P images* (Figure 10D) and the complexity of the frames (Figure 10E). Moreover, given the fact that the specific complexity is similar for frames of different sizes (Figure 10C), it can be expected that overall frame complexity, which is the product of specific frame complexity and frame size, depends on frame size (Figure 10A).

With respect to the unexpected common features, the following findings are noteworthy and relate to previously found regularities in paintings (Redies et al., 2007a,b, 2012; Forsythe et al., 2011; Amirshahi et al., 2012, 2013; Braun et al., 2013):

First, it has been demonstrated that paintings are moderately complex on average (Redies et al., 2012), as exemplified by Berlyne's (1971) u-shaped curve for the effect of complexity on aesthetic preference (see also Forsythe et al., 2011). Strikingly, frames tend to increase the complexity of the *PwF images* compared to images of the paintings alone (*P images*; Figure 5A). As the observer zooms out to observe the paintings in their surround or in museum scenes, complexity decreases again to reach values similar or lower than those of the *P images* (Figure 5A). We conclude that the frame constitutes a *barrier of complexity* that separates the painting from its surround.

Second, results from previous studies suggest that the self-similarity of paintings is relatively high compared to other image categories (Amirshahi et al., 2012, 2013; Redies et al., 2012), and is stable at different levels of the PHOG analysis (Figure 8; Amirshahi et al., 2013). In the present study, *PwF images* have lower self-similarity values than *P images*. This result implies that the PHOG features of the paintings are less similar to the frames than to themselves (Figure 5B). The difference between *P images* and *PwF images* is larger for more self-similar paintings (Figure 11C). When zooming out into the surround and museum scenes, self-similarity of the *PwF/S images* and *MSc images* decreases even further (Figure 5B). Therefore, the frame provides a *transition* from its highly self-similar interior (i.e., the painting) to the less self-similar surroundings of the museum environment.

Third, paintings exhibit a low degree of anisotropy, both in the Fourier domain (Koch et al., 2010; Melmer et al., 2013) and in the PHOG analysis (Amirshahi et al., 2012; Redies et al., 2012), i.e., the measured statistical properties are about equally strong across orientations in artworks. In contrast, luminance gradients of cardinal (horizontal and vertical) orientations predominate in museum architecture and design, as expected. On average, the *PwF images* assume values intermediate between the *P images* and images of the paintings with their surround (*PwF/S images* and *MSc images*; Figure 5C). The frame thus provides a *transition* between the low anisotropy of the paintings and the high anisotropy of the museum environment.

### Methodological considerations

Figures 7, 8 demonstrate that the measures calculated in the present study depend on image size and the level of analysis. However, the above conclusions for the relative differences between image categories are robust, irrespective of image resolution (Figure 7), and for the analysis for self-similarity at levels higher than level 1 (Figure 8).

A shortcoming of our image analysis is that most frames are 3d structures and depth information is lost in the 2d images analyzed. Nevertheless, some information about the 3d shape of the frames can be reconstructed perceptually from shading phenomena, which, in turn, depend on lighting conditions. In the present study, our aim was to study the effect of framing in the normal museum environment. For this reason, we photographed paintings hanging on the wall under the actual lighting conditions in the museums rather than with special illumination.

### Differences between museums

When comparing the three museums, we also found differences between them. Some of the differences may relate to the fact that the museums exhibit paintings from different art periods (Table 2).

Among the three museums, the NRW Collection, which comprises 20th century art, deviates the most. In particular, the average relative frame area is smaller (Figure 4B) and the specific frame complexity is lower (Figure 10B), resulting in lower overall complexity of the frames (Figure 9B), compared to the other two museums. As a consequence, the complexity difference between paintings with and without frames (*PwF images* and *P images*) is smaller, too (Figure 9A). Unlike in the other two museums, there is no relation between frame complexity and the complexity of the framed paintings (Figure 10E) although the frames of more complex paintings tend to be more complex (Figure 10F). Overall, these results suggest that the frames in the NRW Collection have less influence on the statistics of the framed paintings *(PwF images)* than in the two other museums. This difference can hardly be explained by differences in painting size (Figure 4A), but we note that the *P images* of the NRW Collection are less complex on average than in the other two museums (Figure 5A). Complexity is lower also for the *MSc images* (Figure 5A) so that, conceivably, the frames play less of a barrier role than in the other museums.

The differences between the PG and the NG are less pronounced. For paintings of similar size, the frames of the NG are slightly wider than in the PG on average (Figures 4A,B). Complexity, self-similarity and anisotropy are similar in both museums (Figure 5), but frame complexity (Figure 9B) and the difference in complexity between *P images* and *PwF images* (Figure 9A) are higher in the NG. In the PG, more complex paintings tend to have more complex frames on average (Figure 9F).

In previous centuries (Mendgen, 1995; Mitchell and Roberts, 1996; Siefert, 2010) and in other museums (e.g., the Art Historical Museum in Vienna), the hanging of the paintings was and still is more crowded, with paintings hanging close to each other. In future work, it will therefore be of interest to investigate what effect, if any, this hanging practice has on the perceptual properties of the museum environment and possibly on framing. Also, the museum staff may have personal preferences in their frame choice, which may contribute to differences in framing practice. Finally, the present study was restricted to rectangular frames with straight inner borders. The visual properties of museum paintings that are exhibited without frames, with roundish frames or with frames that have with irregular borders, remain to be investigated.

### Comparison of different types of reproduction

Finally, we asked whether the measures investigated in the present study change with the presentation mode of the paintings. This question is important because it has been argued that aesthetic experience depends to a large degree on the circumstances, under which the artworks are presented. For example, the museum atmosphere is thought to sustain aesthetic experience and original artworks are considered to be potentially more impressive than copies of them (see, for example, Danto, 2003). In the present study, we did not find any difference in the calculated values between photographs of the original museum paintings, reproductions scanned from books and images from the Google Art Project. We conclude that images of paintings can have relatively constant statistical image properties, as long as the process of reproduction is of sufficiently high quality. Nevertheless, the changes in these properties that are induced by zooming out from the paintings into the museum environment suggest that the museum is a rather special visual setting. It is likely that this setting differs from that of art books or reproductions displayed on a computer screen. Conceivably, such differences in the visual environment modulate the aesthetic experience of the observer.

### Conclusions

Why are paintings in many museums shown with frames and what is the perceptual effect of the frames on the paintings? Although picture framing is a phenomenon that is common and widespread in many cultures, there have been few, if any, studies on this question to date. We applied modern digital image analysis methods to study frames in three major museums that cover major periods of Western art. We measured higher-order image properties that were previously studied in paintings (complexity, self-similarity and anisotropy) and found common frame characteristics in all three museums as well as differences between museums. In all museums, frames provide a barrier of high complexity between the paintings and the museum environment, and a transition in terms of self-similarity and anisotropy. Besides these universal properties, distinct differences in frame characteristics between the museums possibly reflect variations in frame preference for different art periods. Our study provides the baseline for future scientific investigations on how and why humans use frames to demarcate pictures and to separate them from their visual environment.

### Conflict of interest statement

The authors declare that the research was conducted in the absence of any commercial or financial relationships that could be construed as a potential conflict of interest.
